# Evaluation of colonization with multidrug-resistant microorganisms and the incidence of attributable infections in patients undergoing hematopoietic stem cell transplantation

**DOI:** 10.55730/1300-0144.6148

**Published:** 2025-12-01

**Authors:** Burcu ÇALIŞKAN DEMİRKIRAN, Gülşen İSKENDER, İpek MUMCUOĞLU, Duygu MERT, Sinan DAL, Tuba DAL, Semra TUNÇBİLEK, Mustafa ERTEK, Fevzi ALTUNTAŞ

**Affiliations:** 1 Department of Infectious Diseases and Clinical Microbiology, Dr Abdurrahman Yurtaslan Ankara Training and Research Hospital, Ankara, Turkiye; 2 Department of Medical Microbiology, Dr Abdurrahman Yurtaslan Ankara Training and Research Hospital, Ankara, Turkiye; 3 Division of Hematology, Department of Internal Medicine, Dr Abdurrahman Yurtaslan Ankara Training and Research Hospital, Ankara, Turkiye

**Keywords:** Hematopoietic stem cell transplantation, multidrug-resistant organisms, surveillance culture

## Abstract

**Background/aim:**

The global rise in multidrug-resistant organisms (MDROs) has led to an increased incidence of MDRO infections following hematopoietic stem cell transplantation (HSCT), particularly during the preengraftment period. The objective of this study was to evaluate the relationship between pretransplant colonization by MDROs or carbapenem-resistant *Enterobacterales* (CRE) and the occurrence of infections caused by phenotypically similar microorganisms during the preengraftment period, as well as to assess the clinical outcomes of patients colonized with MDROs prior to transplantation.

**Materials and methods:**

This prospective cohort study included patients diagnosed with hematological malignancies who underwent HSCT at the bone marrow transplant unit of Dr. Abdurrahman Yurtaslan Ankara Oncology Training and Research Hospital between 01 May 2023 and 30 July 2024. Weekly surveillance cultures were obtained at admission and thereafter, including axillary, inguinal, and perianal swabs for MDRO/CRE detection and stool samples for vancomycin-resistant *Enterococcus* (VRE) colonization. The effect of MDRO colonization on the development of infections caused by phenotypically similar MDRO pathogens during the preengraftment period was analyzed.

**Results:**

A total of 118 patients were included in the study. The infection rate with CRE pathogens in patients colonized at least once and in those without CRE colonization was 23.3% and 1.3%, respectively (p = 0.004). The incidences of sepsis (p = 0.003), septic shock (p = 0.01), intensive care unit transfer (p = 0.006), and 28-day mortality (p < 0.001) were significantly higher among patients colonized at admission.

**Conclusion:**

Early detection of CRE colonization through surveillance culture screening may facilitate timely initiation of appropriate antibiotic therapy for CRE infections during the early preengraftment period, potentially improving clinical outcomes and survival.

## 1. Introduction

Infections are a major cause of morbidity and mortality among individuals who have undergone hematopoietic stem cell transplantation (HSCT) [1]. The early preengraftment period represents the time of greatest risk for bacterial infections [2]. In the event of febrile neutropenia during this period, broad-spectrum antipseudomonal antibiotics such as piperacillin–tazobactam, cefepime, or ceftazidime are empirically recommended in current guidelines [3,4]. It is recommended that escalation and deescalation strategies be considered, and that carbapenems be administered to patients with known multidrug-resistant infections, hemodynamic instability, or sepsis. However, with the global rise in antimicrobial resistance, this recommendation may be inadequate in centers with a high incidence of multidrug-resistant organisms (MDROs). Although recent studies have identified MDRO colonization in HSCT recipients as a risk factor for posttransplant MDRO bacteremia, the relationship between colonization and infection with phenotypically similar multidrug-resistant (MDR) pathogens remains unclear [5]. The ECIL-10 guideline recently highlighted that the risk of infection with phenotypically similar organisms increases in patients colonized with MDROs^1^

Awareness of MDRO colonization may serve as an early warning indicator for the development of MDRO infection in HSCT recipients and may guide the timely initiation of optimal antibiotic therapy in the event of infection. Moreover, surveillance cultures for detecting MDRO colonization have been recognized as a valuable tool for preventing and controlling microbial spread in bone marrow transplant units [6]. In accordance with the recommendations of the hospital infection control committee, routine surveillance cultures have been performed on the day of hospitalization and weekly thereafter for patients admitted to our bone marrow transplant unit since January 2023, in response to the rising incidence of MDRO infections.

This study aimed to assess the impact of MDRO or CRE colonization, identified through weekly surveillance cultures, on the development of MDRO or CRE infections during the posttransplant period among HSCT recipients in our bone marrow transplant unit. Additionally, the study sought to evaluate the clinical outcomes of these patients.

## 2. Materials and methods

### 2.1. Study design

This study was a prospective cohort study. The study population comprised adult patients diagnosed with hematological malignancies who underwent HSCT at the bone marrow transplant unit of Dr. Abdurrahman Yurtaslan Ankara Oncology Training and Research Hospital between 01 May 2023, and 30 July 2024. The required sample size was calculated as 100 using G*Power analysis (effect size = 0.283) based on a similar study [7]; however, the study was completed with 118 patients to account for possible exclusions during follow-up [8].

### 2.2. Surveillance culture screening

Patients diagnosed with hematologic malignancies (AML, ALL, MM, lymphoma, etc.) who were followed up in the bone marrow transplant unit with a planned HSCT were included in the study.

In our center, routine surveillance cultures to detect colonization with MDR pathogens have been performed during hospitalization in the bone marrow transplant unit and weekly thereafter since 2023. Swab cultures were obtained from the axillary, inguinal, and perianal regions to detect MDRO and CRE colonization. Stool cultures were performed to detect VRE colonization.

During the posttransplant follow-up period, cultures were obtained from suspected infection sites in cases of fever or clinical suspicion of infection. Demographic data, clinical follow-up findings, and culture results were recorded. The development of MDRO or CRE infections during the posttransplant period was recorded.

### 2.3. Infection control measures

All patients treated in the bone marrow transplantation unit were hospitalized in single rooms equipped with high-efficiency particulate air (HEPA) filtration systems. All patients received two chlorhexidine baths per week in accordance with the standard clinical hospitalization procedures of the bone marrow transplant unit.

Standard isolation precautions—including hand hygiene, appropriate use of personal protective equipment, and equipment decontamination—were implemented in accordance with institutional infection control protocols. In addition, protective (reverse) isolation measures—such as visitor restrictions, mandatory mask use by healthcare personnel, and avoidance of fresh flowers or uncooked fruits and vegetables—were consistently applied for all patients. Patients colonized or infected with multidrug-resistant (MDR) pathogens were additionally placed under contact isolation precautions.

### 2.4. Definitions

MDRO was defined as resistance to three or more antimicrobial classes [9]. The term “carbapenem-resistant *Enterobacterales*” (CRE) refers to members of the order *Enterobacterales* that are resistant to at least one carbapenem antibiotic, including ertapenem, imipenem, or meropenem [10]. Vancomycin-resistant *Enterococcus* (VRE) was defined as resistance to vancomycin (MIC > 4 mg/L) [11].

Febrile neutropenia was defined as a single oral temperature ≥38.3 °C (101 °F) or a temperature ≥38.0 °C (100.4 °F) sustained for at least 1 h in neutropenic patients [12].

The modified early warning score (MEWS) was used to monitor HSCT recipients for early signs of clinical deterioration. MEWS is a physiological scoring system based on a set of routinely measured clinical parameters. Each parameter—including respiratory rate, heart rate, blood pressure, urine output, temperature, oxygen saturation, level of consciousness, and overall clinical concern—is assigned a score ranging from 0 to 3. A MEWS ≥ 4 was considered indicative of a high risk for clinical deterioration, potentially associated with sepsis [13].

The need for vasopressors to maintain a mean arterial pressure ≥65 mm Hg together with a serum lactate level >2 mmol/L was considered indicative of septic shock [14].

### 2.5. Microbiological analysis

Swab samples sent for screening were inoculated onto CHROMID CARBA agar (bioMérieux, Marcy-l’Étoile, France) to detect carbapenem-resistant bacteria. Rectal swab samples for VRE screening were inoculated onto CHROMID VRE agar (bioMérieux, Marcy-l’Étoile, France). The plates were incubated for 24–48 h at 35 ± 2 °C under ambient atmospheric conditions and then examined. Antibiotic susceptibility of the isolated microorganisms was assessed using the disk diffusion method, with incubation at 35± 2 °C for 24 h. The results were interpreted according to the breakpoints defined by the European Committee on Antimicrobial Susceptibility Testing (EUCAST) guidelines^2^

When fever or infection was suspected in HSCT recipients, appropriate clinical specimens—such as blood, urine, sputum, or wound swabs—were collected. The samples were inoculated onto appropriate culture media—including 5% sheep blood agar, chocolate agar, and MacConkey agar (bioMérieux, Marcy-l’Étoile, France)—in accordance with standard microbiological procedures. Blood cultures were processed using automated systems—BACTEC (Becton, Dickinson and Company, Franklin Lakes, NJ, USA) or BacT/ALERT (bioMérieux, Marcy-l’Étoile, France)—and subcultured onto solid media following positive signal detection. All inoculated plates were incubated at 35–37 °C for 18–48 h under aerobic, 5% CO_2_-enriched, or anaerobic conditions, depending on the specimen type and the suspected pathogen. The grown microorganisms were initially evaluated based on colony morphology and Gram staining. Definitive identification was performed using the Vitek MS MALDI-TOF mass spectrometry system (bioMérieux, Marcy-l’Étoile, France). Antimicrobial susceptibility testing was performed using the disk diffusion method, and results were interpreted according to the EUCAST breakpoint guidelines^2^.

### 2.6. Management of febrile neutropenia during follow-up

Febrile neutropenia was defined as a single oral temperature ≥101 °F (38.3 °C) or ≥100.4 °F (38 °C) sustained for at least 1 h, with an absolute neutrophil count (ANC) <500 cells/μL [15]. Patients who developed febrile neutropenia during follow-up received antibiotic therapy within the first hour, in accordance with the institutional management algorithm and international guideline recommendations [16].

When febrile neutropenia occurred in HSCT recipients during follow-up, empirical antimicrobial therapy was initiated according to the institutional treatment algorithm. In cases without known MDR pathogen colonization or infection and with an uncomplicated clinical presentation, an escalation approach using piperacillin–tazobactam or cefoperazone–sulbactam was employed. If no clinical improvement was observed within 72–96 h, empirical carbapenem therapy was continued or combined with an aminoglycoside or colistin. In patients presenting with sepsis or with a documented history of MDR pathogen colonization or infection, an empirical deescalation approach with carbapenem monotherapy or carbapenem combined with an aminoglycoside was initiated. When MDR colonization was known and infection by the colonizing pathogen was suspected, targeted antibiotic therapy was administered according to the corresponding antibiogram results. When culture results were positive during follow-up, antimicrobial regimens were subsequently adjusted according to culture and susceptibility findings.

### 2.7. Assessment of risk factors and clinical outcomes

To identify risk factors for colonization, the following variables were evaluated: hospitalization within the previous 3 months, intensive care unit (ICU) admission within the previous 3 months, history of infections within the previous 3 months, isolation of an MDR pathogen within the past year, receipt of allogeneic HSCT, and carbapenem use within the last month. Clinical outcomes—including the development of sepsis or septic shock, ICU transfer, and 28-day mortality—were evaluated for all patients.

### 2.8. Statistical analysis

Data were analyzed using IBM SPSS Statistics, version 23.0 (IBM Corp., Armonk, NY, USA). The distribution of continuous variables was described by median and interquartile range (IQR), and categorical variables by number and percentage. The normality of continuous variables was evaluated using the Kolmogorov–Smirnov test. The Mann–Whitney U test was used for two-group comparisons of continuous variables. The chi-square test was used to compare categorical variables between groups. To assess the association between MDR pathogen colonization and subsequent infection, relative risk (RR) values and 95% confidence intervals (CIs) were calculated. A p < 0.05 was considered statistically significant.

## 3. Results

A total of 118 adult patients with hematologic malignancies were followed up before and after HSCT during the study period. The median age of the patients was 44.5 years (IQR, 28.75–55), and 72 (61%) were male. The median length of hospitalization was 25 days (IQR, 20.75–28). Among the study population, 33 patients (28%) were diagnosed with AML, 28 (23.7%) with ALL, and 21 (17.8%) with MM. Overall, 73 patients (61.9%) underwent allogeneic HSCT (Table 1).

Febrile neutropenia occurred in 96 (81.4%) patients following transplantation. The sources of infection in febrile neutropenic patients, as determined by clinical, laboratory, and radiologic findings, were identified as follows: bloodstream infections (33 cases, 34.4%), gastrointestinal system infections (including esophagitis, gastroenteritis, and neutropenic enterocolitis; 14 cases, 14.6%), skin and soft tissue infections (10 cases, 10.4%), respiratory system infections (eight cases, 8.3%), urinary tract infections (four cases, 4.2%), and other infections (three cases, 3.1%). In 24 cases (25%), the source of infection remained unidentified (Table 2).

The causative pathogen was isolated in 37 (30.5%) patients. Of these, 15 (40.5%) were MDRO but carbapenem-susceptible *Enterobacterales*, 11 (30%) were CRE, five (13.5%) were Gram-positive cocci, and six (16.2%) were other pathogens. VRE was not identified as the causative pathogen in any case (Table 2).

Among 17 patients colonized with CRE at the time of hospitalization, four (23.5%)—including three bloodstream infections and one urinary tract infection—developed infection with a phenotypically similar pathogen during the early preengraftment period, whereas seven (6.9%) of 101 noncolonized patients developed infection with a phenotypically similar pathogen (p = 0.084). In the cumulative weekly surveillance cultures obtained during hospitalization, 10 (23.2%) of 43 patients colonized with CRE at least once—comprising eight bloodstream infections, one urinary tract infection, and one other infection—developed infection with a phenotypically similar pathogen, whereas only one (1.3%) of 75 noncolonized patients developed infection with a phenotypically similar pathogen (p < 0.05) (Table 3).

There was no statistically significant difference in the development of infection during the preengraftment period among patients colonized with MDRO but carbapenem-susceptible *Enterobacterales* according to surveillance screening results at hospital admission and total weekly surveillance (p = 0.73 and p = 0.66, respectively). VRE colonization was detected in 11 patients at hospital admission and in 27 patients during total weekly surveillance; however, no VRE infections was observed (Table 3).

All patients were hospitalized in single rooms, and standard isolation measures were applied. Patients colonized with MDR pathogens during hospitalization or follow-up were placed under contact isolation, and no MDR pathogen outbreaks occurred in the unit. Compliance with isolation measures and hand hygiene among healthcare workers was verified as 100% by the Infection Control Committee.

Broad-spectrum antipseudomonal antibiotics (piperacillin–tazobactam, cefoperazone–sulbactam, etc.) were initiated in 62 (52.5%) patients, while carbapenems were initiated in 42 (35.6%) patients. In 51 (49%) patients, antibiotic therapy was modified due to persistent fever or lack of clinical improvement during follow-up. Among these, five (9.8%) had ESBL-producing Enterobacteriaceae, and 20 (39.2%) had CRE colonization. Antibiotic therapy was adjusted according to the antibiogram of the colonizing organism, with 22 (21.6%) patients transitioned to carbapenem monotherapy and 27 (26.5%) to carbapenem-based combination therapy. Furthermore, 16 patients (seven with ESBL-producing Enterobacteriaceae and nine with CRE) exhibited growth of resistant Gram-negative bacteria in their cultures.

The rate of carbapenem-resistant *Enterobacterales* colonization was higher in patients with a history of ICU admission within the past 3 months and in those from whom MDR pathogens had been isolated within the previous year, showing statistically significant differences (p < 0.05 and p < 0.05, respectively). Posttransplant follow-up revealed that sepsis, ICU transfer, and 28-day mortality rates were higher in patients colonized with CRE at hospital admission, with statistically significant differences (p < 0.05, p < 0.05, and p < 0.05, respectively). The clinical characteristics and clinical courses of patients with and without carbapenem-resistant *Enterobacterales* colonization are presented in Table 4.

## 4. Discussion

HSCT is an essential therapeutic approach that improves survival in patients with hematologic malignancies. The preengraftment period following transplantation is the phase most frequently associated with bacterial infections and increased mortality [17]. In recent years, infections caused by MDROs have increased worldwide, underscoring the growing clinical challenge of managing resistant infections in HSCT recipients [18].

In our study, based on surveillance culture screenings performed at hospital admission and weekly thereafter, patients who were colonized with CRE at least once during follow-up had a higher rate of CRE infection during the preengraftment period. A statistically significant difference was observed in weekly surveillance results, but not in admission cultures alone. These findings underscore the importance of performing regular weekly surveillance cultures in HCST recipients. Van Leeuwen et al. demonstrated that weekly screening identified new colonizations missed at baseline and facilitated the early implementation of infection control interventions, thereby optimizing antimicrobial management [19]. Kamel et al. demonstrated that active pretransplant screening cultures effectively identified colonization with MDR organisms—including methicillin-resistant *Staphylococcus aureus* (MRSA), CRE, and extended-spectrum β-lactamase (ESBL)-producing bacteria—and predicted the risk of preengraftment infections [20]. Santos et al. reported that colonization by MDR bacteria in HSCT recipients was associated with a higher risk of bloodstream infections and increased mortality [21].

Previous studies have shown that the risk of infection is higher in patients colonized with multidrug-resistant organisms (MDROs) before allogeneic transplantation compared with those undergoing autologous transplantation [22,23]. Consistent with these findings, the present study found a higher prevalence of CRE colonization among allogeneic HSCT recipients. Recognition of pretransplant colonization can guide empiric antibiotic therapy and support the early initiation of appropriate combination regimens when necessary [18].

It is noteworthy that 51 patients (49%) who received empiric antibiotic therapy required treatment modification due to persistent fever and lack of clinical response, with 26.5% switched to carbapenem-based regimens, including combination therapies. Approximately half of these patients (47%) were colonized with a resistant pathogen, and their subsequent therapy was guided by the antibiogram of the colonizing organism. According to the current ECIL-10 guidelines, antibiotic therapy may be adjusted based on the susceptibility profile of the colonizing organism in patients with documented CRE colonization. Tailoring antimicrobial therapy according to colonization data may improve clinical outcomes^3^

The present study did not detect any cases of VRE infection among either colonized or noncolonized patients. According to the current literature, the rate of VRE bacteremia among VRE-colonized patients in our country is approximately 1.8% [24]. The presence of VRE colonization does not necessarily indicate an equivalent risk of infection with the phenotypically similar pathogen as observed in CRE colonization. The low incidence of VRE infection among VRE-colonized patients suggests the feasibility of a patient-based antibiotic management strategy. Previous studies have demonstrated that VRE colonization is a risk factor during the preengraftment period, particularly in allogeneic HSCT recipients [25,26]. A lower risk of infection has been reported in patients undergoing autologous HSCT and in those with solid tumors [27,28]. Implementing a screening program for VRE colonization in centers with a low incidence of VRE infection does not appear to be a cost-effective strategy. Nevertheless, identifying colonization in allogeneic HSCT recipients may contribute to the empirical management of VRE-targeted antibiotic therapy [29,30].

According to the findings of this study, colonization with CRE was observed more frequently in patients who had been admitted to the ICU within the previous 3 months. Previous studies have identified recent hospitalization as a risk factor for colonization with MDR organisms [31].

Implementing preventive measures to reduce colonization may help prevent subsequent infections with resistant pathogens.

The MEWS is a widely used tool for detecting early clinical deterioration in hospitalized patients. Previous studies have shown that MEWS is a sensitive predictor of ICU admission in patients with hematologic malignancies, demonstrating superior performance compared with the quick sequential organ failure assessment score [32]. The practicality of MEWS lies in its simplicity and reliance on routinely measured physiological parameters, allowing for rapid bedside assessment without the need for laboratory data. In the context of HSCT, MEWS may serve as a valuable tool for the early identification of patients at risk for severe infection or sepsis, facilitating prompt clinical intervention and potentially improving outcomes.

Several studies have suggested that pretransplant MDRO colonization is associated with higher mortality; however, other studies have not demonstrated a significant correlation between MDRO colonization and overall survival [33–35]. The present study demonstrated that sepsis, ICU transfer, and 28-day mortality were more common among colonized patients than among those without colonization. Therefore, the results of colonization cultures may serve as predictors of short-term mortality.

The implementation of isolation bundles has been shown to reduce CRE colonization [36]. In this study, all patients were hospitalized in HEPA-filtered single rooms under standard and protective isolation protocols, including contact precautions for CRE-colonized patients. These practices likely contributed to the absence of MDR outbreaks and underscore the importance of strict infection control measures in reducing colonization and subsequent infection.

## 5. Limitations

The main limitation of this study is the relatively small sample size. However, its prospective design constitutes a methodological strength. Although the difference was not statistically significant, MDR-colonized patients at admission exhibited a higher rate of MDR infection during follow-up, which may be attributable to the limited number of colonized patients included in the study. Nevertheless, weekly surveillance screenings revealed that MDR-colonized patients had a significantly higher rate of MDR infection development.

Genotypic identification to determine whether infections caused by multidrug-resistant (MDR) pathogens originated from colonizing strains was not performed, as genetic sequencing of the isolates was not available at our center. Nevertheless, demonstrating infections caused by phenotypically similar organisms with comparable antimicrobial susceptibility profiles may still yield valuable insights for antimicrobial stewardship and infection control strategies.

Another limitation of this study is the considerable heterogeneity among patients in terms of the type of hematopoietic stem cell transplantation (HSCT) received and the underlying hematologic malignancies. Both factors could substantially influence clinical outcomes, pathogen diversity, and infection risk. This heterogeneity should be taken into account when interpreting the study findings.

## 6. Conclusion

In conclusion, routine surveillance culture screening for CRE in HSCT recipients may facilitate early and appropriate antibiotic therapy, thereby supporting optimal posttransplant infection management and improving clinical outcomes.

## Figures and Tables

**Table 1:** Demographic characteristics of hematopoietic stem cell transplantation (HSCT) recipients.

Variables	n	%
Sex (female/male)	72/46	61/39
Age, median (Q1–Q3)	44.5 (28.75–55)	
Length of hospital stay, median (Q1–Q3)	25 (20.75–28)	
Types of transplantation		
Autologous	45	39.1
Allogeneic	73	61.9
Diagnosis		
Acute myeloid leukemia	33	28
Acute lymphoblastic leukemia	28	23.7
Multiple myeloma	21	17.8
Diffuse large B cell lymphoma	11	9.3
Hodgkin lymphoma	10	8.6
Other non-Hodgkin lymphoma	9	7.2
Aplastic anemia	1	0.8
Chronic myelomonocytic leukemia	1	0.8
Myelodysplastic syndrome	1	0.8
Plasma cell leukemia	1	0.8
Plasmacytoma	1	0.8
Total	118	100

**Table 2:** Febrile neutropenia and infection among hematopoietic stem cell transplantation (HSCT) recipients during the preengraftment period.

Variables	Noncolonized n = 55		CRE colonized n = 43		MDR, but carbapenem susceptible colonized N = 15		VRE colonized n = 27		Total	
	n	%	n	%	n	%	n	%	n	%
**Presence of febrile neutropenia**										
No	12	21.8	9	20.9	1	93.3	2	7.4	22	19.6
Yes	43	78.2	34	79.1	14	6.7	25	92.6	96	81.4
**Source of infection**										
Bloodstream	12	27.9	17	50	4	28.6	11	44	33	34.4
Gastrointestinal system	9	20.9	5	14.7	3	21.4	2	8	17	17.7
Skin and soft tissue	6	14	2	5.9	2	14.3	1	4	10	10.4
Respiratory system	4	9.3	3	8.9	2	14.3			8	8.3
Urinary tract	1	2.3	1	2.9	1	7.1	1	4	4	4.2
Unknown origin	11	25.6	6	17.6	2	14.3	10	40	24	25
Total	43	100	34	100	14	100	25	100	96	100
**Presence of culture growth**										
No	43	78.2	22	51.2	11	73.3	14	56	82	69.5
Yes	12	21.8	21	48.8	4	26.7	11	44	37	30.5
**Isolated microorganism**										
MDR, but carbapenem sensitive *Enterobacterales*	7	58.4	6	28.6	2	50	4	36.4	15	40.5
Carbapenem resistant *Enterobacterales*	1	8.3	10	47.6	0	0	4	36.4	11	30
Gram positive cocci	1	8.3	2	9.5	2	50	2	18.1	5	13.5
Other	3	25	3	14.3	0	0	1	9.1	6	16.2
Total	12	100	21	100	4	100	11	100	37	100

* Abbreviations: CRE: carbapenem-resistant *Enterobacterales*; MDR: multidrug-resistant; VRE: vancomycin-resistant *Enterococcus*.

**Table 3:** Screening results and infection status of hematopoietic stem cell transplantation (HSCT) recipients during hospitalization and weekly surveillance cultures. (Boldface type denotes statistical significance.)

Variables	Colonization status at the time of admission			Colonization status based on the total results of weekly screening		
	Infection/colonization	Infection/noncolonization	p	Infection/colonization	Infection/noncolonization	p
Carbapenem-resistant *Enterobacterales*						
n	4/17	7/101	0.084	10/43	1/75	**0.004**
%	23.5	6.9		23.2	1.3	
MDRO, but carbapenem susceptible *Enterobacterales*						
n	0/1	12/117	0.73	2/15	10/103	0.66
%	0	10.3		13.3	9.7	
VRE						
n	0/11	0/107	-	0/27	0/91	-
%	0	0		0	0	

**Table 4:** Conditions associated with colonization by carbapenem-resistant *Enterobacterales* (CRE) at hospital admission and related clinical outcomes.

Variables	Noncolonized n = 101		Colonized with CRE n = 17			
	n	%	n	%	RR (95%CI)	p**
Hospitalization in the last 3 months	80	79.2	16	94.1		0.26
ICU admission in the last 3 months	0	0	4	23.5		<0.001
History of infections in the last 3 months	39	38.6	10	58.8		0.11
MDR pathogen isolation in the last 1 year	6	5.9	7	41.2	1.9 (1–3.5)	<0.001
Presence of allogeneic HCST	58	57.4	15	88.2	1.2 (1.0–1.3)	0.016
Carbapenem use in the last month	9	8.9	4	23.5		0.07
Clinical outcome						
Sepsis	14	13.9	8	47.1	1.4 (1–1.9)	0.003
Septic shock	7	6.9	5	29.4	1.5 (0.9–2.4)	0.01
Transfer to ICU	5	5	5	29.4	1.7 (0.9–3.3)	0.006
28-day mortality	4	4	5	29.4	2 (0.9–4.1)	<0.001

* Abbreviations: CRE: carbapenem-resistant *Enterobacterales*; HSCT: hematopoietic stem cell transplantation; ICU: intensive care unit; MDR: multidrug-resistant.

** Relative risk (RR) and 95% confidence interval (CI) were calculated using 2 × 2 contingency tables and analyzed with the chi-square test.

## Footnotes

1 Averbuch D, Guisado MA, Akova M, Baccelli F, Blijlevens N et al. (2024) ECIL-10 Bacterial: febril neutropenia- duration of therapy-new drugs. [online]. Website https://www.ecil-leukaemia.com/images/ecil-10/ECIL_10_-_bacterial_group_final_recommendations.pdf [accessed 15 December 2024].

2 The European Committee on Antimicrobial Susceptibility Testing. Breakpoint tables for interpretation of MICs and zone diameters. Version 14.0 2024 (2024) [online]. Website http://www.eucast.org [accessed 15 December 2024].

3 Averbuch D, Guisado MA, Akova M, Baccelli F, Blijlevens N et al. (2024) ECIL-10 Bacterial: febril neutropenia- duration of therapy-new drugs. [online]. Website https://www.ecil-leukaemia.com/images/ecil-10/ECIL_10_-_bacterial_group_final_recommendations.pdf [accessed 15 December 2024].

## References

[1] Gea-Banacloche J Risks and epidemiology of infections after hematopoietic stem cell transplantation Ljungman P Snydman D Boeckh M Transplant Infections Springer Cham 2016 15 81 99 10.1007/978-3-319-28797-3_6

[2] Almyroudis N Fuller A Jakubowski A Sepkowitz K Jaffe D Pre-and post-engraftment bloodstream infection rates and associated mortality in allogeneic hematopoietic stem cell transplant recipients Transplant Infectious Disease 2005 7 1 11 17 10.1111/j.1399-3062.2005.00088.x 15984943

[3] Klastersky J De Naurois J Rolston K Rapoport B Maschmeyer G ESMO Guideline Committee. Management of febrile neutropaenia: ESMO clinical practice guidelines Annals of Oncology 2016 27 111 118 10.1093/annonc/mdw325 27664247

[4] Averbuch D Orasch C Cordonnier C Livermore DM Mikulska M ECIL4, a joint venture of EBMT, EORTC, ICHS, ESGICH/ESCMID and ELN. European guidelines for empirical antibacterial therapy for febrile neutropenic patients in the era of growing resistance: summary of the 2011 4th European Conference on Infections in Leukemia Haematologica 2013 98 12 1826 1835 10.3324/haematol.2013.091025 24323983 PMC3856957

[5] Kropshofer G Hetzer B Knoll M Meryk A Salvador C What we learn from surveillance of microbial colonization in recipients of pediatric hematopoietic stem cell transplantation Antibiotics 2022 12 1 2 10.3390/antibiotics12010002 36671203 PMC9854581

[6] Tacconelli E Mazzaferri F de Smet AM Bragantini D Eggimann P ESCMID-EUCIC clinical guidelines on decolonization of multidrug-resistant gram-negative bacteria carriers Clinical Microbiology and Infection 2019 25 7 807 817 10.1016/j.cmi.2019.01.005 30708122

[7] Mendes ET Salomao MC Tomichi LM Oliveira MS Graca M Effectiveness of surveillance cultures for high priority multidrug-resistant bacteria in hematopoietic stem cell transplant units Revista do Insttituto Medicina Tropical de Sao Paulo 2021 63 77 10.1590/S1678-9946202163077 PMC8580484 34755816

[8] Faul F Erdfelder E Lang A-G Buchner A G*Power 3: A flexible statistical power analysis program for the social, behavioral, and biomedical sciences Behavior research methods 2007 39 2 175 191 10.3758/bf03193146 17695343

[9] Magiorakos A-P Srinivasan A Carey RB Carmeli Y Falagas M Multidrug-resistant, extensively drug-resistant and pandrug-resistant bacteria: an international expert proposal for interim standard definitions for acquired resistance Clinical microbiology and infection 2012 18 3 268 281 10.1111/j.1469-0691.2011.03570.x 21793988

[10] Tamma PD Aitken SL Bonomo RA Mathers AJ van Duin D Infectious Diseases Society of America 2023 Guidance on the Treatment of Antimicrobial Resistant Gram-Negative Infections Clinical Infectious Diseases 2024 428 481 10.1093/cid/ciae403 37463564

[11] Giske CG Turnidge J Cantón R Kahlmeter G EUCAST Steering Committee Update from the European Committee on Antimicrobial Susceptibility Testing (EUCAST) Journal of Clinical Microbiolology 2022 60 3 276 321 10.1128/JCM.00276-21 PMC8925892 34346716

[12] Freifeld AG Bow EJ Sepkowitz KA Boeckh MJ Ito JI Clinical practice guideline for the use of antimicrobial agents in neutropenic patients with cancer: 2010 update by the Infectious Diseases Society of America Clinical infectious diseases 2011 52 4 56 93 10.1093/cid/cir073 21205990

[13] Subbe CP Kruger M Rutherford P Gemmel L Validation of a modified Early Warning Score in medical admissions OJM: An International Journal of Medicine 2001 94 10 521 526 10.1093/qjmed/94.10.521 11588210

[14] Evans L Rhodes A Alhazzani W Antonelli M Coopersmith CM Surviving sepsis campaign: international guidelines for management of sepsis and septic shock 2021 Critical care medicine 2021 49 11 1063 1143 10.1007/s00134-021-06506-y 34605781

[15] Freifeld AG Bow EJ Sepkowitz KA Boeckh MJ Ito JI Infectious Diseases Society of America. Clinical practice guideline for the use of antimicrobial agents in neutropenic patients with cancer: 2010 update by the infectious diseases society of america Clinical Infectious Diseases 2011 52 4 56 93 10.1093/cid/cir073 21205990

[16] Averbuch D Orasch C Cordonnier C Livermore DM Mikulska M European guidelines for empirical antibacterial therapy for febrile neutropenic patients in the era of growing resistance: summary of the 2011 4th European Conference on Infections in Leukemia Haematologica 2013 98 12 1826 1835 10.3324/haematol.2013.091025 24323983 PMC3856957

[17] Sahin U Toprak SK Atilla PA Atilla E Demirer T An overview of infectious complications after allogeneic hematopoietic stem cell transplantation Journal of Infection and Chemotherapy 2016 22 8 505 514 10.1016/j.jiac.2016.05.006 27344206

[18] Alp S Akova M Antibacterial resistance in patients with hematopoietic stem cell transplantation Mediterranean journal of hematology and infectious diseases 2017 9 1 1 14 10.4084/MJHID.2017.002 PMC5224809 28101308

[19] Van Leeuwen LPM du Toit J McMillan B Tadzimirwa GY Oosthuizen J Bloodstream infections and colonization in hematopoietic stem cell transplant recipients at a south african center: a retrospective analysis Transplant Cell Therapy 2025 31 4 269 282 10.1016/j.jtct.2025.02.010 39952366

[20] Kamel NA Abdalla MS Al Ali A Alshahrani MY Aboshanab KM Effectiveness of pre-transplant screening for high-priority multidrug-resistant pathogens on pre-engraftment infections after hematopoietic stem cell transplantation Infection and Drug Resistance 2024 17 2249 2260 10.2147/IDR.S463868 38854781 PMC11162205

[21] Santos ES Lima ACM Breda GL Tomaz APdO Nabhan SK Colonization by multidrug-resistant bacteria in hematological patients undergoing hematopoietic stem cell transplantation and clinical outcomes: a single-center retrospective cohort study Transplant Infectious Disease 2023 25 5 1398 1408 10.1111/tid.14119 37561358

[22] Krawiec KM Strzałka P Stańczak K Czemerska M Szmigielska A Evaluation of colonization and infection profile in allogeneic hematopoietic stem cell transplantation recipients Acta Haematologica Polonica 2024 55 2 112 122 10.5603/ahp.98671

[23] Patriarca F Cigana C Massimo D Lazzarotto D Geromin A Risk factors and outcomes of infections by multidrug-resistant gram-negative bacteria in patients undergoing hematopoietic stem cell transplantation Biology of Blood and Marrow Transplantation 2017 23 2 333 339 10.1016/j.bbmt.2016.11.005 27826061

[24] Kaya A Kaya SY Balkan II Bayramlar OF Mete B Risk factors for development of vancomycin-resistant enterococcal bacteremia among VRE colonizers: a retrospective case control study Wiener klinische Wochenschrift 2021 133 478 483 10.1007/s00508-020-01733-7 32910333

[25] Kamboj M Cohen N Huang Y-T Kerpelev M Jakubowski A Impact of empiric treatment for vancomycin-resistant *Enterococcus* in colonized patients early after allogeneic hematopoietic stem cell transplantation Biology of Blood and Marrow Transplantation 2019 25 3 594 598 10.1016/j.bbmt.2018.11.008 30448456 PMC6445740

[26] Benamu E Deresinski S Vancomycin-resistant *Enterococcus* infection in the hematopoietic stem cell transplant recipient: an overview of epidemiology, management, and prevention F1000Research 2018 7 3 24 10.12688/f1000research.11831.1 29333263 PMC5750719

[27] Rolston K Jiang Y Matar M VRE fecal colonization/infection in cancer patients Bone marrow transplantation 2007 39 9 567 568 10.1038/sj.bmt.1705639 17351646

[28] Ford C Lopansri B Gazdik M Snow G Webb B The clinical impact of vancomycin-resistant *Enterococcus* colonization and bloodstream infection in patients undergoing autologous transplantation Transplant Infectious Disease 2015 17 5 688 694 10.1111/tid.12433 26256692

[29] Kang Y Vicente M Prasad S Brielmaier B Pisano J Evaluation of risk factors for vancomycin-resistant *Enterococcus* bacteremia among previously colonized hematopoietic stem cell transplant patients Transplant Infectious Disease 2013 15 5 466 473 10.1111/tid.12120 23911080

[30] Weinstock DM Conlon M Iovino C Aubrey T Gudiol C Colonization, bloodstream infection, and mortality caused by vancomycin-resistant *Enterococcus* early after allogeneic hematopoietic stem cell transplant Biology of Blood and Marrow Transplantation 2007 13 5 615 621 10.1016/j.bbmt.2007.01.078 17448922

[31] Cardoso T Ribeiro O Aragão IC Costa-Pereira A Sarmento AE Additional risk factors for infection by multidrug-resistant pathogens in healthcare-associated infection: a large cohort study BMC infectious diseases 2012 12 1 9 10.1186/1471-2334-12-375 23267668 PMC3566942

[32] Van Mourik N Oomen JJ van Vught LA Biemond BJ van den Bergh WM The predictive value of the modified early warning score for admission to the intensive care unit in patients with a hematologic malignancy-A multicenter observational study Intensive Critical Care and Nursing 2023 79 103486 10.1016/j.iccn.2023.103486 37441816

[33] Scheich S Lindner S Koenig R Reinheimer C Wichelhaus TA Clinical impact of colonization with multidrug-resistant organisms on outcome after allogeneic stem cell transplantation in patients with acute myeloid leukemia Cancer 2018 124 2 286 296 10.1002/cncr.31045 28960264

[34] Scheich S Reinheimer C Brandt C Wichelhaus TA Hogardt M Clinical impact of colonization with multidrug-resistant organisms on outcome after autologous stem cell transplantation: a retrospective single-center study Biology of Blood and Marrow Transplantation 2017 23 9 1455 1462 10.1002/cncr.31045 28528711

[35] Forcina A Lorentino F Marasco V Oltolini C Marcatti M Clinical impact of pretransplant multidrug-resistant gram-negative colonization in autologous and allogeneic hematopoietic stem cell transplantation Biology of Blood and Marrow Transplantation 2018 24 7 1476 1482 10.1016/j.bbmt.2018.02.021 29501780

[36] Hayden MK Lin MY Lolans K Weiner S Blom D Prevention of colonization and infection by Klebsiella pneumoniae carbapenemase–producing Enterobacteriaceae in long-term acute-care hospitals Clinical Infectious Diseases 2015 60 8 1153 1161 10.1093/cid/ciu1173 25537877 PMC8381216

